# Nephrin Regulates Lamellipodia Formation by Assembling a Protein Complex That Includes Ship2, Filamin and Lamellipodin

**DOI:** 10.1371/journal.pone.0028710

**Published:** 2011-12-14

**Authors:** Madhusudan Venkatareddy, Leslie Cook, Kamal Abuarquob, Rakesh Verma, Puneet Garg

**Affiliations:** Division of Nephrology, University of Michigan Medical School, Ann Arbor, Michigan, United States of America; Thomas Jefferson University, United States of America

## Abstract

Actin dynamics has emerged at the forefront of podocyte biology. Slit diaphragm junctional adhesion protein Nephrin is necessary for development of the podocyte morphology and transduces phosphorylation-dependent signals that regulate cytoskeletal dynamics. The present study extends our understanding of Nephrin function by showing in cultured podocytes that Nephrin activation induced actin dynamics is necessary for lamellipodia formation. Upon activation Nephrin recruits and regulates a protein complex that includes Ship2 (SH2 domain containing 5′ inositol phosphatase), Filamin and Lamellipodin, proteins important in regulation of actin and focal adhesion dynamics, as well as lamellipodia formation. Using the previously described CD16-Nephrin clustering system, Nephrin ligation or activation resulted in phosphorylation of the actin crosslinking protein Filamin in a p21 activated kinase dependent manner. Nephrin activation in cell culture results in formation of lamellipodia, a process that requires specialized actin dynamics at the leading edge of the cell along with focal adhesion turnover. In the CD16-Nephrin clustering model, Nephrin ligation resulted in abnormal morphology of actin tails in human podocytes when Ship2, Filamin or Lamellipodin were individually knocked down. We also observed decreased lamellipodia formation and cell migration in these knock down cells. These data provide evidence that Nephrin not only initiates actin polymerization but also assembles a protein complex that is necessary to regulate the architecture of the generated actin filament network and focal adhesion dynamics.

## Introduction

The renal glomerulus forms the filtering unit in the kidney and is comprised of a tuft of capillaries that are covered on the urinary side by glomerular visceral epithelial cells or podocytes. Podocytes are unique epithelial cells with a large cell body and long processes that branch and engulf the capillaries. The primary processes arise from the cell body and further divide to form secondary and tertiary processes. Microtubules and intermediate filaments form the framework for the primary and secondary processes whereas the tertiary or *foot processes* are rich in actin. The foot processes of neighboring podocytes interdigitate and form a specialized intercellular junction called the *slit diaphragm*. Podocytes play a key role in maintaining the selective filtration barrier by preventing the passage of cellular elements and large macromolecules from the blood into the urinary space. Glomerular diseases that present with protein leak in the urine are almost always associated with podocyte foot process spreading or effacement. Podocyte *effacement* may occur due to either developmental defects or injury to a mature podocyte and is a direct consequence of altered slit diaphragm structure and cytoskeletal changes of the foot processes. There is a direct relationship between the tertiary structure of the podocyte and a healthy podocyte intercellular junction. This is supported by the identification of human mutations in slit diaphragm junctional proteins Nephrin (Nphs1) and Podocin (Nphs2) [1], [2] where the morphology of the podocyte is altered. Nephrin is a transmembrane protein belonging to the immunoglobulin superfamily, and is targeted to the podocyte intercellular junction. During development Nephrin expression coincides with emergence of the nascent processes at the basolateral aspect of the podocyte [3]. Human mutations in Nephrin [2] or deletion of Nephrin in mice [4], [5] results in protein leak and developmental failure of foot process formation. Podocyte development, effacement and repair require robust cytoskeletal changes that involve actin dynamics. It is not surprising that actin regulation has been the focus of numerous investigations, which attempt to integrate the intercellular junctional dynamics and the three dimensional architecture of the podocyte. Initial observations revealed Nephrin's ability to recruit adaptor proteins that regulate actin polymerization. Following the Src kinase *Fyn* dependent tyrosine phosphorylation of Nephrin's cytoplasmic domain [6], there is recruitment of several Src homology 2 (Sh2) domain containing adaptor proteins including Nck1/2, Crk, phospholipase Cγ and the p85 subunit of PI3 kinase [7], [8], [9], [10], [11]. Nephrin has since been shown to associate with other proteins belonging to the actin polymerization machinery including Arp2/3, nWASp, Synaptopodin, ZO-1, IQGAP1 and CD2ap [12], [13], [14]. Furthermore, in a cell culture model, activation of the Nephrin-Neph1 complex by itself induces actin filament nucleation and elongation [7], [8], [15], [16].

Actin filaments not only provide the structural framework for cells but are also essential for a variety of cellular processes like cell movement, cell division, cellular trafficking of cargo and organelles and cell junction formation. Cells use several mechanisms to generate actin filaments locally at the membrane in response to external signals. In this regard, the phosphoinositol signaling cascade plays an important role in response to an external signal and is considered to be one of the initial events to occur proximal to the membrane. In order to form an actin filament, cells need to not only initiate actin polymerization (nucleate) but also maintain the filament by controlling depolymerization [17], [18]. Beyond nucleation and maintenance of the filament, controlled branching and cross-linking is also essential to generate a complex structure such as lamellipodia [19], [20] at the leading edge. Lamellipodia are believed to be the actual motor that pulls the cell forward or propels a membrane protrusion. Focal adhesions are sites of contact between the cell and its underlying substrate or matrix. Thus, lamellipodia formation and migration requires an active dialogue between the sites of actin polymerization and the focal adhesion complex to regulate its turnover allowing the cell to propel itself in the direction of the signal [21].

Cognizant of the role of Nephrin in development of the podocyte foot process intercellular junction, we hypothesized that Nephrin recruits a protein complex that not only regulates nucleation of actin filaments but regulates generation of an actin network resulting in lamellipodia formation. In an initial screen, we observed binding of Ship2 to Nephrin in a tyrosine phosphorylation dependent manner. Ship2 is a ubiquitously expressed Sh2 domain containing inositol phosphatase [22], [23], [24]. The closely related homologue ship1 is primarily expressed in hematopoetic tissues [25]. Ship2 contains an N-terminal Sh2 domain, a catalytic 5′ phosphatase domain and a proline rich domain containing a NPXY motif that binds to phosphotyrosine binding (PTB) domains [22]. The phosphoinositides play a vital role in cell motility signaling, Ship2 regulate PI3 kinase mediated events in response to a transmembrane signal by catalyzing the removal of 5-phosphate from PI(3,4,5)P3, producing PI(3,4)P2 [26], [27]. It has also been demonstrated that Ship2 localizes to focal adhesions and lamellipodia and interacts with the cytoskeletal proteins Filamin and Vinexin [22], [28]. In enteropathogenic *E. coli* (EPEC) and *Vaccinia* virus, Ship2 mediates development of actin pedestals [29], [30] in response to Ship2 mediated generation of PI(3,4)P2 and enrichment of Lamellipodin at the sites of actin polymerization. Lamellipodin (LPD) is a ubiquitous protein and as its name suggests is important in lamellipodial dynamics. LPD acts as a scaffold to assemble a complex of proteins necessary for focal adhesion dynamics, including Ena/VASP.

Here we report that upon activation, Nephrin recruits Ship2 in a p21 activated serine threonine kinase (Pak1) dependent manner. Ship2 is further able to enrich Filamin and Lamellipodin at sites of actin polymerization resulting in increased formation of lamellipodia. As a consequence of its interaction with the described protein complex, Nephrin is able to influence lamellipodia formation and focal adhesion dynamics.

## Experimental Procedures

### Antibodies

Purified rabbit polyclonal antibodies against Nephrin [3], and phospho-Nephrin [7] were previously described. Antibodies against Ship2, p-Ship2 (Y1135), Filamin, p-Filamin (S2152) were obtained from Cell Signal (Danvers, MA). Anti-CD16 (clone 3G8) antibody (BD Bioscience, San Jose, CA); rhodamine and dylight-conjugated goat anti-mouse IgG (Pierce, Rockford, IL); Alexa Fluor 594 goat anti-rabbit IgG (Invitrogen, Carlsbad, CA); GST-HRP, β-actin and Flag antibody were obtained from Sigma-Aldrich (St. Louis, MO). 50A9 antibody against the human nephrin extracellular domain was a gift from K. Tryggvason [31]. Lamellipodin (Santa Cruz Biotechnology, Santa Cruz, CA) and Pak1 (Cell Signal, Danvers, MA) antibodies were also obtained commercially.

### Plasmids

Plasmids encoding GFP-Ship2 and HA-Ship2 were obtained as a gift from Christina A. Mitchell, Monash University, Clayton, Victoria, Australia, YFP-Lamellipodin was a gift from Frank Gertler, Massachusetts Institute of Technology, Boston, USA. 3× Flag-Nephrin was generated by sub-cloning human Nephrin into pCMV-SC-CF (Agilent) plasmid. Pak1 K299R (#12210) and Pak1 P13A (#12207), Filamin A S2152 (# 8983) were obtained from Addgene (www.addgene.com). DsRed- Filamin A was a gift from Fumi Nakamura at Harvard University, Boston, Massachusetts, USA. Mammalian plasmids encoding mouse Nephrin [3], Fyn [32], human Nephrin was a gift from K. Tryggvason [31] were described previously. The CD16-HA construct was a gift from B. Mayer (University of Connecticut, Farmington, CT) [33]. Constructs encoding a fusion protein consisting of the CD16 extracellular domain, CD7 transmembrane domain and Nephrin cytoplasmic domain and the mutants were generated using PCR based techniques. Tapp1 plasmid was obtained from Open Biosystems (Huntsville, AL) and was sub-cloned into pTag-YFP-C plasmid (Evrogen, Moscow, Russia) using standard PCR method. Restriction digestion and DNA sequencing were used to confirm all construct sequences.

### Immunoprecipitation and Immunoblotting

Proteins were extracted from plasma membranes in RIPA buffer (PBS containing 0.1% SDS, 1% Nonidet P-40, 0.5% sodium deoxycholate and 100 mM potassium iodide). Endogenous immunoprecipitation was performed by extracting tissue in RIPA buffer containing 0.1% BSA.

### Cell culture

Transient transfections were carried out in human podocyte (Hupo) cells (gift from Moin Saleem) cultured in RPMI with glutamax (Invitrogen, Carlsbad, CA) supplemented with 10% FBS (Invitrogen Corp.) 200 U/ml penicillin and streptomycin (Roche Applied Science, Indianapolis, IN), and ITS (Insulin, Transferrin and Selenium) (Invitrogen, Carlsbad, CA). M2 Melanoma cell line was a kind gift from Paul Janmey (University of Pennsylvania, Philadelphia, USA) [34].Transfections were performed using Lipofectamine 2000 (Invitrogen Corp.), Fugene HD (Roche, Indianapolis, IN) and electroporation using Amaxa nucleofactor II (Lonza biosystem, Walkersville, MD) as per manufacturer's directions. For 50A9 antibody studies, human podocytes expressing human 3×Flag-Nephrin were serum starved for 30 minutes prior to treatment with 50A9 mab (10 µg/ml) in complete media and incubated for 30 minutes at 4°C. Cells were washed and treated with anti-mouse IgG (20 µg/ml) for 5 minutes at 37°C and lysed in RIPA buffer.

### Knock-Down Cell Lines

Human podocytes were infected using at least five different shRNA for Ship2 and Lamellipodin each packaged in lentivirus (Sigma Mission shRNA, St. Louis, MO). Pak1 knock down was performed using shRNA cloned in pGIPz Lentiviral vector (Open Biosystems, Huntsville, AL) that also expresses GFP under a bicistronic promoter to mark cells expressing the shRNA. Non-silencing pGIPz vector (R4346) was used for control infection (Open Biosystems, Huntsville, AL). Cells with stable integration were selected using puromycin dihydrochloride (Sigma, St. Louis, MO) at a concentration of 2 µg/ml. Knock-down was verified by western blot. Cells with no evidence of knock-down were used as controls. We have created numerous human podocytes knock down cell lines in our lab. In the case of Ship2 there was evidence of knock down with all five shRNA. As control we used cell lines generated previously with no evidence of knock down of ship2 using similar shRNA constructs obtained from Sigma (See Table 1 for sequence of shRNA used for knock down).

**Table 1:** ShRNA sequence for knock down of human Ship2 , Lamellipodin and Pak1.

Ship2 shRNA1	CCGGCCACCCAAGAACAGCTTCAATCTCGAGATTGAAGCTGTTCTTGGGTGGTTTTTG
Ship2 shRNA2	CCGGCCTGAACTACATCAGCAGGAACTCGAGTTCCTGCTGATGTAGTTCAGGTTTTTG
Ship2 shRNA3	CCGGCCGGATTCTGTGGAAATCCTACTCGAGTAGGATTTCCACAGAATCCGGTTTTTG
Ship2 shRNA4	CCGGGACTACCTGAAAGGCAGCTATCTCGAGATAGCTGCCTTTCAGGTAGTCTTTTTG
Ship2 shRNA5	CCGGGCAGCTCAATGCCTTTGACATCTCGAGATGTCAAAGGCATTGAGCTGCTTTTTG
LPD shRNA1	CCGGGCCAGGACTATCGGAACAAATCTCGAGATTTGTTCCGATAGTCCTGGCTTTTTG
LPD shRNA2	CCGGGCACCTTGAAAGGATTATCTTCTCGAGAAGATAATCCTTTCAAGGTGCTTTTTG
LPD shRNA3	CCGGGCTTATATTTATGGAGCGTATCTCGAGATACGCTCCATAAATATAAGCTTTTTG
LPD shRNA4	CCGGGTTGCTCTCTTATGTCTTTATCTCGAGATAAAGACATAAGAGAGCAACTTTTTG
LPD shRNA5	CCGGGCGTCAAATCACAGAAACGAACTCGAGTTCGTTTCTGTGATTTGACGCTTTTTG
Pak1 shRNA1	AGGCCTAGACATTCAAGACAAATAGTGAAGCCACAGATGTATTTGTCTTGAATGTCTAGGCCG
Pak1 shRNA2	CCCAAGAAAGAGCTGATTATTTAGTGAAGCCACAGATGTAAATAATCAGCTCTTTCTTGGGC
Pak1 shRNA3	GCCCGGACCACGGGTGACATATAGTGAAGCCACAGATGTATATGTCACCCGTGGTCCGGGC
Pak1 shRNA4	GGGCATCATGGCCATCGAAATTAGTGAAGCCACAGATGTAATTTCGATGGCCATGATGCCCA
Pak1 shRNA 5	CGCCATCAAGGCTCTGAAGAATAGTGAAGCCACAGATGTATTCTTCAGAGCCTTGATGGCGA

### Puromycin Aminonucleoside Nephrosis

Female Sprague Dawley rats weighing 200–250 g were injected with puromycin aminonucleoside (Sigma-Aldrich, St. Louis, MO) (10 mg/100 g) or PBS (vehicle) intra-peritoneally. Rats were sacrificed and glomeruli isolated using graded sieving as described previously [14].

### Mouse Kidney Perfusion

Perfusion of mouse kidneys with protamine sulfate was carried out as described previously (23, 41). Briefly, C57Bl6 mice were anesthetized with pentobarbital; mouse core temperature was monitored with a rectal probe, and animals were maintained at 37°C throughout the procedure using a heating pad apparatus. Kidneys were perfused with solutions maintained at 37°C through the left ventricle at a pressure of 70 mmHg and an infusion rate of 10 ml/min (42). Perfusion was carried out with PBS for 2 min, followed by perfusion with protamine sulfate (2 mg/ml in PBS; Sigma) for 15 min. For effacement recovery, following perfusion with protamine sulfate, mice were perfused with heparin sulfate (800 g/ml in PBS; Sigma) for 15 min (41). Glomeruli were isolated using graded sieving as described previously [14]. All animal experiments were approved by the University Committee on the Use and Care of Animals Institutional Review Board at the University of Michigan Medical School (Protocol approval # 07683). All work was conducted in accord with the principles and procedures outlined in the National Institutes of Health Guidelines for the Care and Use of Experimental Animals.

### Protein Pull-down

Purified His-tag proteins bound to Talon magnetic beads (Invitrogen Corp, Carlsbad, CA) were incubated with purified GST recombinant proteins where indicated. After washing extensively with PBS containing 0.1% Tween-20, 1 mM sodium orthovanadate, and 1 mM sodium fluoride, protein complexes were eluted, resolved by SDS-PAGE in replicates and immunoblotting with the indicated antibodies.

### CD16/CD7/Neph1 chimera, recruitment experiments

Human Podocyte cells were transfected with CD16/CD7 chimeric constructs bearing HA, Nephrin CD (cytoplasmic domain) and various mutants at the C-terminal end. Thirty hours following transfection, RPMI medium was removed and replaced with fresh medium containing 1 µg/ml CD16 antibody (clone 3G8, Beckman Coulter). Cells were maintained on ice for 1 h. Cells were then washed twice with PBS, 1 µg/ml rhodamine-conjugated or unlabeled anti-mouse IgG (Pierce Biotechnology, Rockford, IL) was added to the media, and incubation was continued at 37°C for 30 min. Cells for immunofluorescence were washed 3 times with PBS and fixed with cytoskeleton buffer (10 mM 2-CN-morpholino-ethanesulfonic acid, 138 mM KCl, 3 mM MgCl_2_, 2 mM EGTA, and sucrose to a final concentration of 0.32 M). On the day of use, 20% paraformaldehyde was added to cytoskeleton buffer stock to achieve a final concentration of 4%. Cover slips were mounted on glass slides using ProLong Gold antifade reagent (Invitrogen Corp, Carlsbad, CA). Samples were analyzed by fluorescence confocal microscopy with an Olympus FV-500 microscope using a 100× oil immersion objective lens and Fluoview software (version TIEMPO 4.3; Olympus). Images were processed using Adobe Photoshop software. All images were acquired at 1024×1024 pixel resolution.

### Cell migration studies using blind well chamber

We used blind well chambers from Neuro- Probe Inc. (Gaithersburg, MD) for these studies. In a BW25 blind well chamber 25 µl of 50A9 mab in serum free media (50 µg/ml) was pipetted into the lower chamber to create a slight positive meniscus. A 12 µm membrane was carefully placed over the filled well to avoid trapping air bubbles at the interface. After screwing in the well retainer, the upper chamber of the well was filled with 100 µl of the indicated cell suspension (2000 cells/ml). We used an incubation time of 30 minutes in our experiments as initial pilot experiments demonstrated migration of cells in control well (mouse IgG) beyond 2 hr of incubation at 37°C. Following incubation the filter paper was carefully removed using a forceps. The side facing the upper wall was cleaned with a cotton swab. The filter paper was then placed on a glass slide and fixed using ProLong Gold antifade reagent with DAPI (Invitrogen Corp, Carlsbad, CA). Cells were counted using an inverted immunofluorescence microscope (Olympus).

## Results

### The SH2 domain containing 5′ inositol phosphatase Ship2 associates with Nephrin in a tyrosine phosphorylation dependent manner

**Figure 1:**
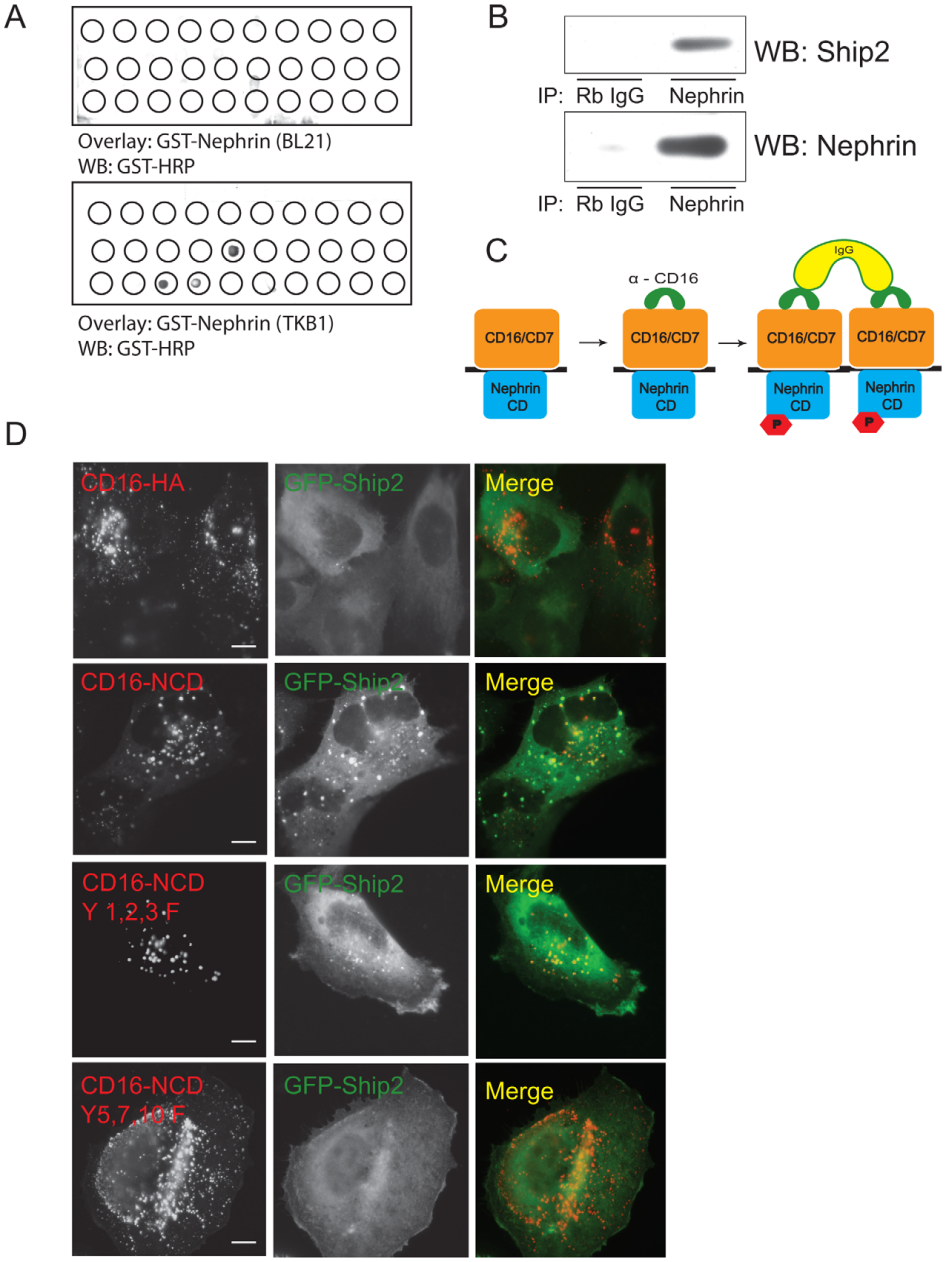
Nephrin interacts with Ship2. ***A*** **.** *Biased Screen*. A library of *his*-tagged Sh2 domains of Sh2-Sh3 domain containing proteins were blotted onto a membrane. The membrane was incubated with either tyrosine phosphorylated (TKB1) or non-phosphorylated (BL21) GST-Nephrin cytoplasmic domain and then probed with horseradish peroxidase-conjugated anti-GST antibody. This figure depicts a representative experiment. ***B.*** *Nephrin and Ship2 associate in vivo.* Co-immunoprecipitation experiment performed on rat glomerular lysates using antibody against Nephrin. ***C.*** *Schematic showing CD16 clustering system*. Chimeras with CD16 extracellular domain, CD7 transmembrane domain, and Nephrin cytosplasmic domain are clustered by the addition of anti-CD16 antibody (primary) and subsequently anti-mouse IgG (secondary), resulting in phosphorylation of Nephrin tyrosine residues. ***D.*** *Ship2 is recruited to CD16/CD7/Nephrin CD clusters at the plasma membrane.* Human podocytes expressing CD16/CD7 chimeric proteins (red) and GFP-Ship2 (green) were treated with anti-CD16 antibody (primary) and rhodamine-conjugated anti-mouse IgG (secondary), fixed, and examined by confocal microscopy. CD16-HA represents a CD16/CD7 chimera in which the Nephrin cytosplasmic domain is replaced by an HA tag and serves as control. CD16/CD7/Nephrin CD chimeras with tyrosine to phenylalanine mutations (Y to F) at indicated residues were used to map the tyrosine responsible for the Nephrin-Ship2 interaction. Data are representative of four separate experiments. Scale bar: 10 µm.

To identify proteins that interact with Nephrin, we generated a library of *His*-tagged Sh2 domains from proteins that contain Sh2-Sh3 domains. The recombinant proteins were purified using cobalt beads (Pierce biotechnology, Rockford, IL) and verified on SDS page gels. The Sh2 domain recombinant proteins were blotted on to a PVDF membrane using a dot blot apparatus. Using a far western technique the membrane was incubated with GST-Nephrin expressed in either BL21 (unphosphorylated) or TKB1 (phosphorylated) *E. coli* as described previously [15], [16]. The membrane was then blotted with GST-HRP antibody (Figure 1A). Using this biased approach we identified Ship2 as one of the Sh2 domain containing proteins that binds to tyrosine phosphorylated Nephrin. We were further able to confirm this association by co-immunoprecipitation of endogenous Nephrin and Ship2 from rat glomerular lysates (Figure 1B). Even though there was a direct interaction between the Ship2 Sh2 domain and Nephrin in the initial screen, a direct interaction between full-length recombinant Ship2 and Nephrin could not be performed. Later findings confirmed that the interaction between Nephrin and Ship2 is indirect. It is likely that the initial findings were due to a promiscuous interaction between the smaller Ship2 Sh2 domain fragment and recombinant GST-Nephrin. To further investigate the interaction between Nephrin and Ship2 we used a model system that recapitulates the consequences of Nephrin ligand binding employed by us and other investigators [7], [8], [15], [16] (see Figure 1C). Fusion proteins were generated that contain the CD16 extracellular domain, CD7 transmembrane domain and Nephrin cytoplasmic domain (or HA tag as control). Addition of anti-CD16 (mouse) antibody (primary or aggregating antibody) to the media of the transfected cells followed by anti-mouse IgG (secondary) leads to tyrosine phosphorylation of the clustered CD16 chimeric proteins at the cell surface which can then be visualized by confocal microscopy [7], [8], [16], [35]. Using the CD16 chimeric model system we observed recruitment of GFP-Ship2 to CD16-Nephrin clusters at the membrane and not to CD16-HA (control) clusters (Figure 1D).

To further map the tyrosine residues responsible for this interaction, we employed several point and compound mutations of tyrosine to phenylalanine (Y to F) residues in the Nephrin cytoplasmic domain. We have previously shown that Nephrin tyrosine residues Y1,2,3 are necessary for recruitment of p85 subunit of PI3 kinase to Nephrin [12]. Nephrin Y5,7,10 residues are important for Nephrin-Nck interaction [4], [5]. We expected Nephrin Y1,2,3 tyrosine residues to be important for interaction with Ship2, based on the role of Ship2 in dephosphorylating PI3 kinase-generated PI(3,4,5)P3 to PI(3,4)P2. However, there was recruitment of GFP-Ship2 to CD16-Nephrin clusters with the Y1,2,3F mutation, the recruitment was abrogated when Nephrin Y5,7,10F mutation was expressed (see Table 2 for Nephrin tyrosine sequences and alternate numbering). Data regarding other tyrosine residue mutations in the Nephrin cytoplasmic domain that did not abrogate Ship2 recruitment are not shown.

**Table 2:** Tyrosine Residues of Mouse Nephrin cytoplasmic domain.

Residue	Sequence	Alternate Tyrosine Numbering in text and Figures
Y1128	YEES	Y1
Y1153	YYSN	Y2
Y1154	YYSN	Y3
Y1172	YRQA	Y4
Y1191	YDEV	Y5
Y1198	YGPP	Y6
Y1208	YDEV	Y7
Y1216	YDLR	Y8
Y1225	YEDP	Y9
Y1232	YQDV	Y10

### Pak1 is recruited to clustered Nephrin in an Nck dependent fashion and results in recruitment of Filamin-Ship2 complex

**Figure 2:**
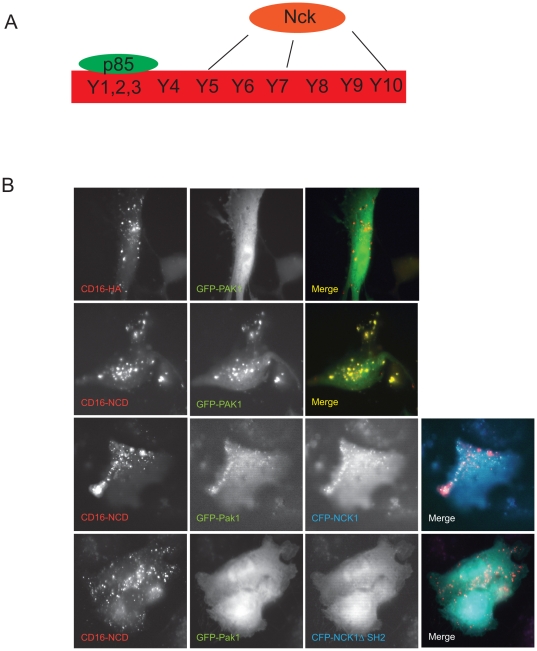
Nephrin recruits p21 activated kinase (Pak1). ***A*** **.** *Nephrin tyrosine residues and known interactions*. Schematic of mouse nephrin cytoplasmic domain highlighting the tyrosine residues and known interacting partners. Phosphorylation on Nephrin tyrosine residues 1, 2 and 3 have been shown to be important for association with the p85 subunit of PI3 kinase. Similarly, Y5,7,10 residues are responsible for Nephrin-Nck interaction. ***B.*** *Nephrin recruits Pak1 in an Nck dependent manner*. Human podocytes expressing the indicated plasmids were clustered as described and examined using confocal microscopy. Nephrin co-localized with Pak1 in the presence of full length Nck. This interaction was abrogated when Nck lacking its Sh2 domain (NckΔSH2) was expressed. Scale bar: 10 µm.

**Figure 3:**
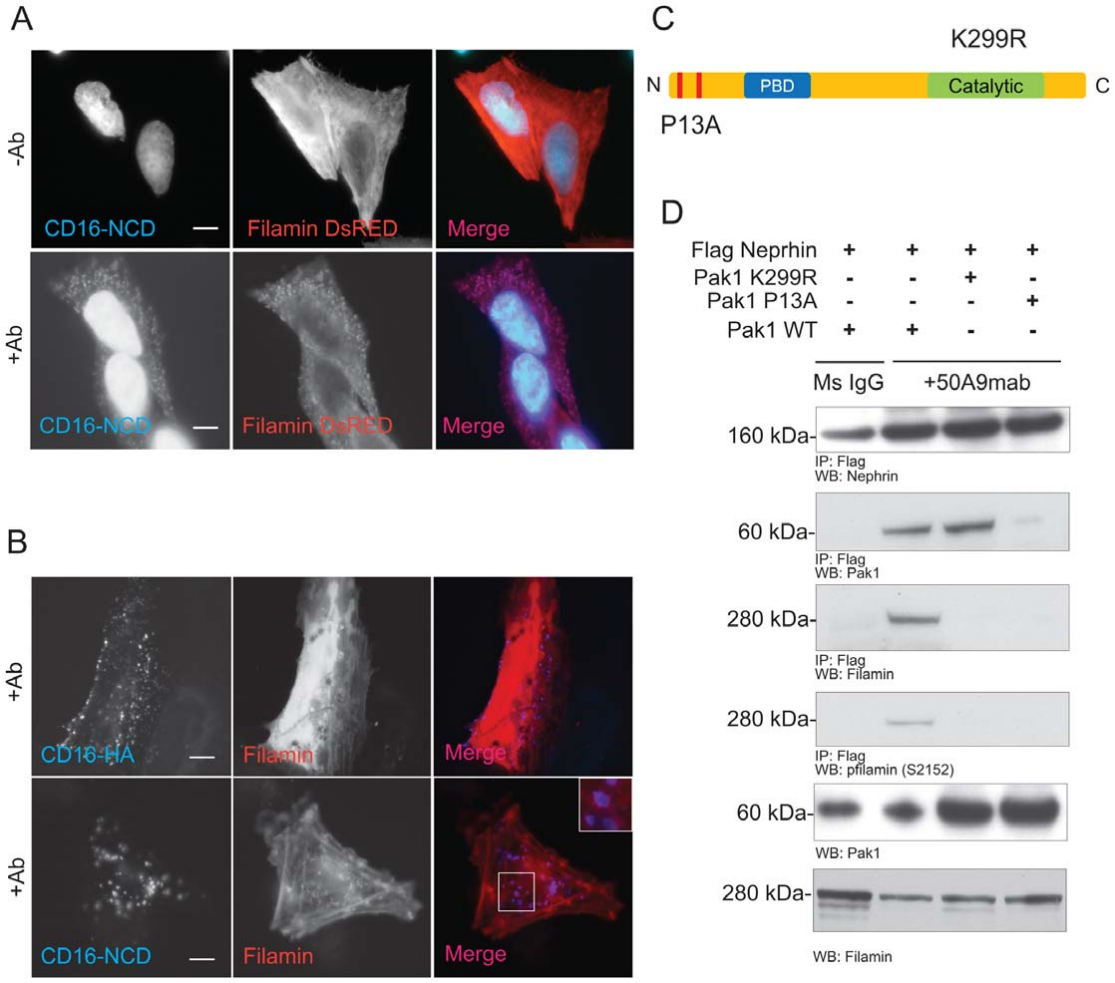
Nephrin interacts with Filamin. ***A***. *Filamin is recruited to CD16-Nephrin clusters at the membrane*. Human podocytes expressing the DsRed-Filamin and CD16-Neprhin were clustered as described and examined using confocal microscopy. DsRed-Filamin is recruited to CD16 clusters (blue) in the presence of the anti-CD16 (primary) antibody (lower panel). **B**. *Endogenous Filamin co-localizes with CD16-Nephrin clusters at the membrane*. Human podocytes expressing CD16-HA and CD16-Nephrin were clustered as described and examined by confocal microscopy. Filamin was detected with rabbit polyclonal anti-Filamin antibody and an Alexa Fluor 594-labeled secondary antibody. Inset in the bottom panel represents a five-fold enlargement of a portion of the micrograph demonstrating co-localization of endogenous Filamin with the CD16-Nephrin cluster. **C**. *Pak1 domain structure.* Pak1 has an N-terminal proline rich region, a p21 rho binding domain (PBD) domain and a C-terminal catalytic domain. The mutation in the proline rich region (P13A) prevents interaction of Pak1 with SH3 domain of adaptor proteins and the K299R mutation disrupts the catalytic activity of Pak1. **D**. *Nephrin activation results in Pak1 dependent phosphorylation of Filamin*. Human podocytes expressing indicated plasmids were treated with 50A9 anti-Nephrin antibody followed by anti-mouse IgG. Lysates were immunoprecipitated with pre-immune sera or Flag antibody. Lysates were run on a separate gel. *WB*, Western blot; *IP*, immunoprecipitation. Scale bar: 10 µm.

In unstimulated cells Ship2 is localized both in the cytoplasm and enriched along the leading edge. It forms a stable complex with Filamin in the cytosol and this interaction determines the membrane localization of Ship2 [28], [36]. Furthermore, Filamin interacts with p21 activated kinase 1 (Pak1) following stimulation of cells by physiological signaling molecules like heregulin [37]. Pak1 is a serine threonine kinase that is important for regulation of the actin cytoskeleton and ruffle formation. The ability of Pak1 to form membrane ruffles is dependent on Filamin as it is only observed in Filamin expressing cells [37]. It is also known that Pak1 interacts with Nck via a proline rich region at its amino terminal. To explain the abrogation of Ship2 recruitment to CD16-Nephrin clusters with the Y5,7,10F mutation, we hypothesized that the Nephrin-Ship2 interaction is preceded by Nephrin-Nck-Pak1 interaction. Following Nck binding to Nephrin Y5,7,10 tyrosine residues, Pak1 is recruited and binds to Nck. This interaction further results in Pak1 mediated recruitment of the Filamin-Ship2 complex. To prove our hypothesis we first investigated whether Pak1 was recruited to Nephrin using the CD16 model and if Nck was necessary for this interaction (See Figure S1 for Nck recruitment to CD16-Nephrin clusters). We were able to demonstrate recruitment of GFP-Pak1 to clustered CD16-Nephrin (Figure 2B) in transfected cultured podocytes. This recruitment was abrogated when a plasmid encoding Nck lacking its Sh2 domain (NCKΔSH2) was expressed (Figure 2B, lower panel) as this prevented Nephrin-Nck association. Using the CD16 model system we were also able to demonstrate recruitment of both transfected DsRed-Filamin (Figure 3A) and endogenous Filamin (Figure 3B) to the CD16-Nephrin clusters at the membrane. Filamin co-localization at the membrane was abrogated in the absence of the aggregating antibody or when CD16-HA was clustered. In order to confirm the necessity of Pak1 in recruitment of Filamin to the CD16-Nephrin clusters, we generated a Pak1 knock-down human podocyte cell line (Pak1^kd^ Hupo) using shRNA constructs that simultaneously express GFP. Knock-down of Pak1 was confirmed by western blotting (Figure S2A). Consistent with our hypothesis, we observed decreased co-localization of Filamin with CD16-Nephrin clusters in cells where Pak1 was knocked down (Figure S2B). To investigate whether the Nephrin-Ship2 interaction required the kinase activity of Pak1, we transiently expressed Pak1 and its mutants along with 3×Flag-hNephrin in human podocytes. The cells were lysed in RIPA buffer following incubation with 50A9 mab (anti-human Nephrin) or mouse IgG as control. As anticipated, Nephrin and Pak1 co-immunoprecipitated when Nephrin was activated using 50A9 mab. We also observed co-immunoprecipitation of endogenous Filamin with Flag-Nephrin only in the presence of wild type Pak1. Additionally, this interaction resulted in phosphorylation of Filamin on S2152, which has been reported to be a PAK1 target by Vadlamudi et al [37]. This phosphorylation site is believed to be important for both Filamin activity [37], [38] and its association with its binding partners which includes integrin. Nephrin-Filamin interaction and Filamin S2152 phosphorylation were abrogated with the expression of a kinase dead Pak1 (Pak1 K299R) or Pak1 plasmid containing a mutation in its proline rich region (Pak1 P13A) which prevents Pak1-Nck binding (Figure 3C). Collectively, these studies demonstrate that Nephrin is able to recruit and phosphorylate Filamin in a Pak1 dependent manner. It also confirms our hypothesis that the Nephrin-Ship2 interaction is preceded by a signaling cascade involving Nck-Pak1 binding at Nephrin's Y5,7,10 tyrosine residues.

### Nck and Filamin are necessary for Ship2 recruitment to clustered Nephrin

**Figure 4:**
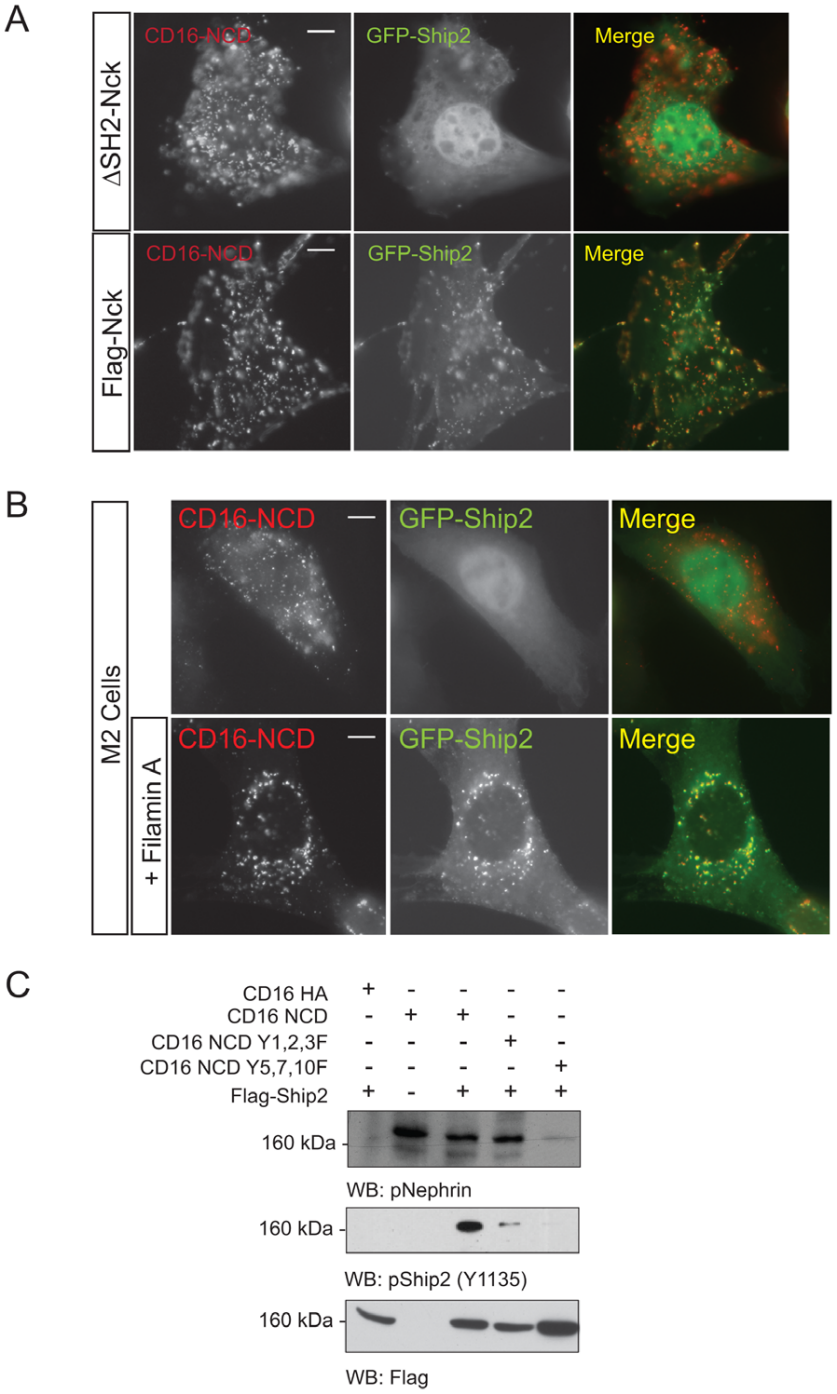
Nck and Filamin are necessary for Nephrin-Ship2 interaction. ***A*** **.** *Ship2 is recruited to CD16-Nephrin clusters in the presence of Nck*. Human podocytes expressing CD16-Nephrin chimeras and GFP-Ship2 were clustered in the presence of either full length Nck or Nck lacking its Sh2 domain (NckΔSH2). ***B.*** *Filamin is required for Ship2 recruitment to CD16-Nephrin clusters*. Human melanoma cells (M2) which lack Filamin were transfected with CD16-Nephrin chimera and GFP-Ship2 and clustered as described and examined by confocal microscopy. WT-Filamin (wild type) was transfected to rescue the M2 cell line. **C.** *Nephrin activation results in Ship2 phosphorylation*. Human podocytes expressing Flag-Ship2 were co-transfected with plasmids encoding the indicated CD-16 chimeric proteins. Cell lysates were resolved by SDS-Page and transferred to a PVDF membrane. The membrane was blotted with a p-Nephrin antibody that recognizes Y5 and Y7 phosphorylation and a p-Ship2 (Y1135) antibody. Scale bar: 10 µm.

To extend our findings we investigated the necessity of Nck and Filamin in the Nephrin-Ship2 interaction. To investigate whether binding of Nck to Nephrin is necessary for Ship2 recruitment we examined co-localization CD16-Nephrin and GFP-Ship2 in the presence of full-length flag tagged Nck and NckΔSH2. GFP-Ship2 was recruited only when full length Nck was expressed (Figure 4A). Similarly, to assess the role of Filamin in Nephrin mediated Ship2 recruitment we used the Filamin deficient human melanoma (M2) cell line. M2 cell lines do not express detectable Filamin mRNA or protein [39]. Using the CD16 system, GFP-Ship2 recruitment was not observed when CD16-Nephrin expressed in M2 cells was clustered (Figure 4B). This phenotype was reversed on expression of Filamin A, suggesting that Ship2 recruitment to Nephrin is also dependent on the presence of Filamin (see Figure S3A for levels of Filamin expression in M2 cells, Human podocytes and M2 cells with Filamin overexpression). Similarly, Dyson et al [28], reported that membrane localization of Ship2 was dependent on Filamin expression when cells were stimulated with EGF. Collectively these results suggest that step-wise signaling is required for Ship2 recruitment to the Nephrin clusters. Nephrin activation results in Nck recruitment and binding to 5,7,10 tyrosine residues of the Nephrin cytoplasmic domain. This further results in Pak1 binding to Nck via its proline rich region. Pak1 then phosphorylates Filamin on its S2152 residue resulting in recruitment and phosphorylation of Ship2.

### Nephrin engagement results in Ship2 tyrosine phosphorylation

The activation of Ship2 has been associated with phosphorylation of Ship2 tyrosine residues. Ship2 undergoes Src Kinase dependent tyrosine phosphorylation on Y986–987 during attachment and spreading of cells [40]. Phosphorylation on Ship2 Y1135 by an unidentified kinase has been observed in cancer cells and is associated with increased Ship2 activity [22], [24]. We hypothesized that Nephrin activation would result in Ship2 tyrosine phosphorylation. Using the CD16 clustering model we observed Ship2 Y1135 phosphorylation in cell culture. This was abrogated when the CD16-Nephrin chimera with Y5,7,10F mutation was clustered (Figure 4C).

### Nephrin activation results in generation of phosphoinositol (3,4) P2 at the membrane and recruitment of Lamellipodin

**Figure 5:**
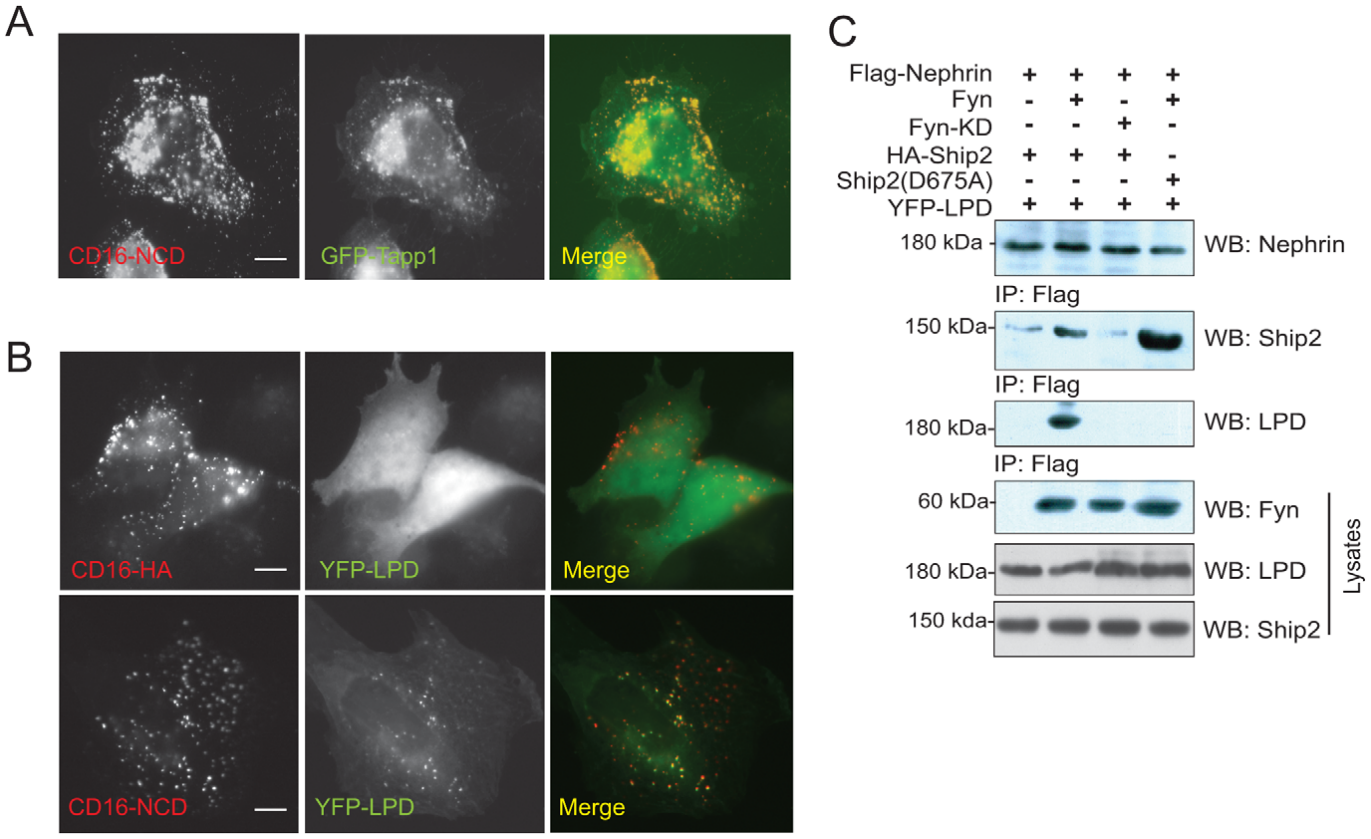
Nephrin activation results in Lamellipodin recruitment. ***A.*** *Nephrin ligation results in generation of P(3,4)P2*. Tapp1 PH domain is a specifically binds to P(3,4)P2. Human podocytes expression CD16-Nephrin and GFP-Tapp1 was clustered as described. There was recruitment of GFP-Tapp1 to CD16 Nephrin clusters suggesting generation of P(3,4)P2 proximal to the clusters at the membrane. ***B.*** *Recruitment of Lamellipodin (LPD)*. Human podocytes expressing GFP-LPD were transfected with the indicated CD16-chimeric proteins. There was recruitment of GFP-LPD to CD16-Nephrin clusters but not to CD16-HA (control). ***C.*** *Nephrin associates with Lamellipodin in a Ship2 dependent manner*. Human podocytes expressing Flag-Nephrin were transfected with the indicated plasmids. Nephrin-LPD co-immunoprecipitation was evident only in presence of wild type Ship2. This was abrogated on transfecting a phosphatase dead mutant of Ship2 (D675A). Scale bar: 10 µm.

Upon activation, Ship2 dephosphorylates phosphoinositol (3,4,5) P3 by removing the phosphate on position 5 to generate PI(3,4)P2. These two 3′phosphoinositides account for less than 0.25% of all inositol containing lipids making them prime candidates for regulatory signaling molecules [41]. The PH domain of TAPP1 binds specifically to PI(3,4)P2 and therefore TAPP1 can be used as a marker for the presence of PI(3,4)P2 [42]. We examined whether there was generation of PI(3,4)P2 using the CD16 clustering model system by demonstrating recruitment of GFP-Tapp1 to the clusters (Figure 5A). PI(3,4)P2 has also been identified as the primary phosphoinositol that binds to the PH domain of Lamellipodin. LPD belongs to the Ena/VASP family of proteins. It acts as a scaffold protein to assemble a protein complex involved in lamellipodia formation and focal adhesion dynamics, and it also promotes axonal guidance in response to slit and netrin signaling [43], [44]. LPD contains six FP4 motifs and binds to Ena/VASP proteins which are regulators of cytoskeletal dynamics that influence the density of the actin network and frequency and persistence of lamellipodia [43]. Using the CD16 system we were able to demonstrate recruitment of YFP-LPD to CD16-Nephrin clusters (Figure 5B). These findings suggest the ability of Nephrin to generate PI(3,4)P2 presumably in a Ship2 dependent manner and recruit LPD.

### Lamellipodin recruitment to Nephrin clusters is dependent on Nephrin mediated activation of Ship2 phosphatase activity

To test whether Ship2 recruitment and activity following Nephrin activation is necessary for LPD recruitment, we transfected a phosphatase deficient mutant of Ship2 (D675A) along with YFP-LPD in cells expressing 3×Flag tagged Nephrin. WT-Fyn or Fyn KD (kinase dead) were expressed as indicated. We were able to co-immunoprecipitate Nephrin and LPD only in the presence of wild type Ship2. While the interaction between Nephrin and Ship2 was maintained in the presence of Ship2 (D675A) mutant (Figure 5C), LPD did not co-immunoprecipitate with Nephrin in the presence of Ship2 (D675A). Similarly, expression of Fyn KD also abrogated the Nephrin-Ship2 and Nephrin-LPD interaction. These observations suggest that both Nephrin tyrosine phosphorylation and Ship2 phosphatase activity are required for LPD recruitment to Nephrin.

### There is increased Interaction of Nephrin with Ship2, Filamin and Lamellipodin following protamine sulfate mediated podocyte injury

**Figure 6:**
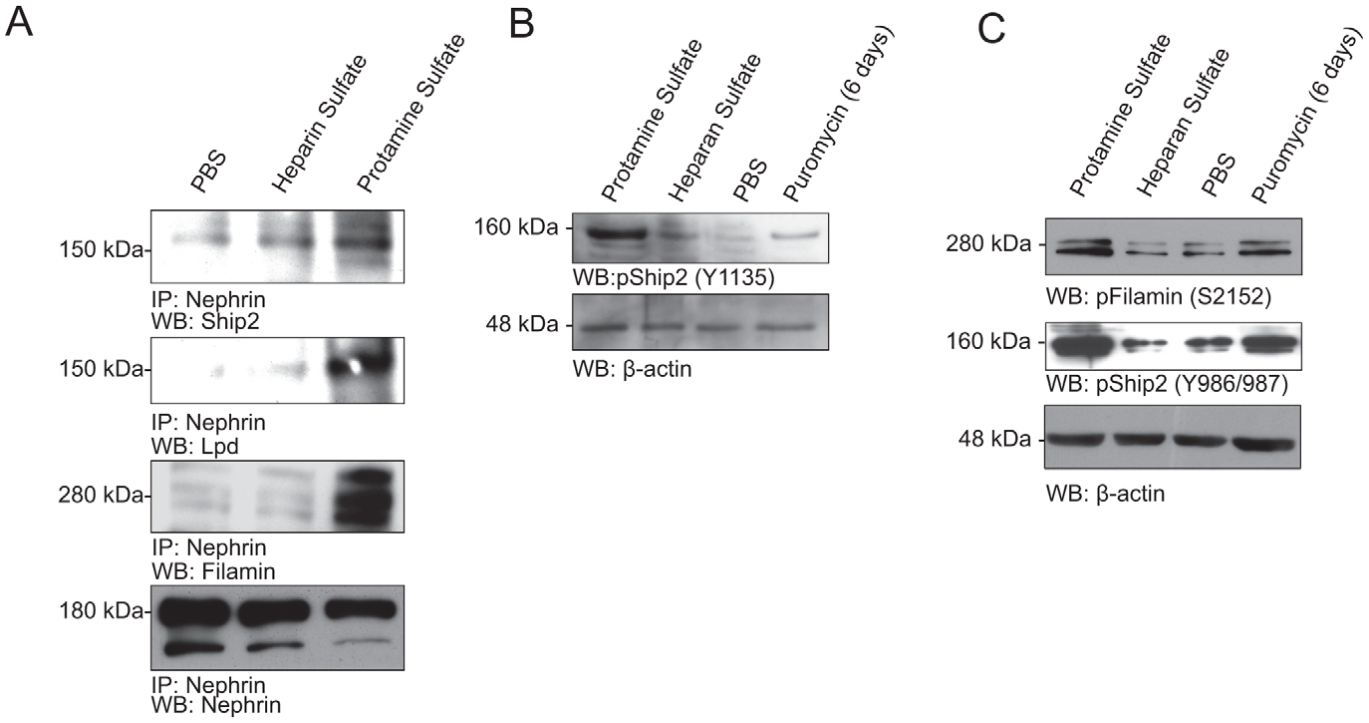
There is increased association of Nephrin with Ship2, Filamin and Lamellipodin following podocyte injury *in vivo*. **A.** *Increase association of Nephrin with Ship2, Filamin and Lamellipodin in protamine sulfate injury model*. Glomerular lysates form mouse perfused with protamine sulfate, protamine sulfate followed by heparin sulfate and PBS (con) were immunoprecipitated with Nephrin antibody and blotted for Ship2, Filamin and Lamellipodin. **B and C.** *There is increased Ship2 and Filamin phosphorylation following podocyte injury*. There is increase in Ship2^Y1135^ and Ship2^Y986/987^ and Filamin^S2152^ phosphorylation in glomerular lysates from PAN (puromycin aminonucleoside) and protamine sulfate treated animals. Glomerular lysates from rats injected with PBS (control) or PAN were lysed and immunoblotted as shown. Similarly, mice were injected with protamine sulfate and heparan sulfate where indicated. *WB*, Western blot; *IP*, immunoprecipitation.

The protamine sulfate model of podocyte injury was employed to examine the interaction of Nephrin with Ship2, Filamin and Lamellipodin in vivo. This model results in rapid foot process spreading concomitant with Nephrin tyrosine phosphorylation [7], [45]. Three month old wild type mice were perfused via the renal artery with either protamine sulfate or control buffer (PBS). Another group of mice were initially perfused with protamine sulfate followed by heparin sulfate which reverses the phenotype. Glomeruli were isolated and lysed in RIPA buffer as described previously [7]. We observed increased association of Nephrin with Ship2, Filamin and Lamellipodin on podocyte injury. This was abrogated after perfusion with heparin sulfate (Figure 6A). This supports the hypothesis that Nephrin phosphorylation in response to injury results in assembly of a protein complex that enables formation of lamellipodia that are reminiscent of foot process effacement.

### Podocyte Injury results in increased phosphorylation of Ship2 and Filamin

To examine phosphorylation of Ship2 and Filamin in an in vivo model, we used glomerular lysates from animals treated with puromycin and protamine sulfate. Glomerular lysates from mice perfused with protamine sulfate showed increased phosphorylation of Ship2 (Y1135) (Figure 6B), Ship2 (Y986/987) and Filamin (S2152) when compared to control mice that were perfused with PBS (Fig. 6C) or heparan sulfate. Similarly, glomerular lysates from rats 6 days following treatment with puromycin aminonucleoside showed increased Ship2 (Y1135), Ship2 (Y986/987) and Filamin (S2152) phosphorylation. These observations suggest that Ship2 and Filamin are activated in response to glomerular injury; presumably facilitating dramatic actin remodeling that occurs during foot process spreading.

### Ship2 and Filamin knock down result in aberrant actin tail morphology and decreased efficiency of lamellipodia formation

**Figure 7:**
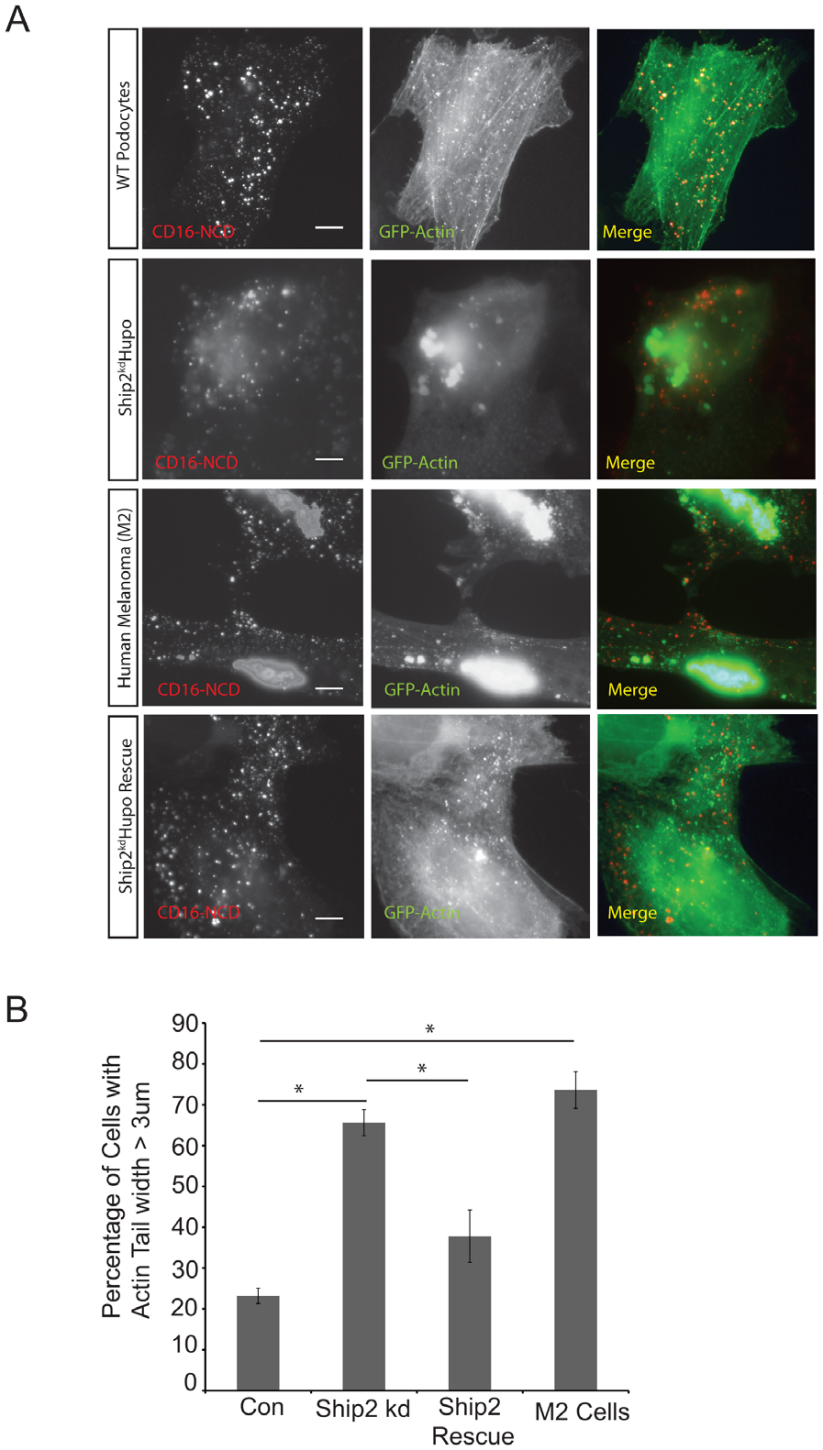
Ship2 knock down and lack of Filamin results in aberrant actin tails. ***A.*** Human podocytes WT or Ship2^kd^ Hupo expressing the indicated plasmids were clustered as described and examined using confocal microscopy. M2 cells which lack Filamin were used to examine the actin tail formation in the absence of Filamin. ***B*** **.** Percentage of transfected cells with actin tails wider than 3 µm. Most cells with aggregated actin were easily identifiable. Image J software (National Institute of Health) was used to determine the width of the actin tails. Approximately 100 cells were analyzed in each condition. Data are representative of five different experiments. Scale bar: 10 µm.

Phosphoinositides are ubiquitous membrane components and serve to localize signaling proteins including actin regulatory proteins to the inner wall of the membrane following receptor mediated signaling [46]. Nephrin activation or ligand binding results in the formation of actin pedestals or tails seen as discrete foci of actin polymerization that localize with clustered CD16-Nephrin [7], [8], [35]. These foci are reminiscent of a burst of actin polymerization observed when pathogens like enteropathogenic *E.coli*, *Vaccinia* and *Listeria* hijack the host actin polymerization machinery to move around the cell [47], [48], [49], [50]. We hypothesized that actin tail morphology and lamellipodia formation would be altered in the absence of Ship2 or if Nephrin mediated Ship2 activation was perturbed. A human podocyte cell line was prepared in which Ship2 was stably knocked down (Ship2^kd^ Hupo) using lentivirus encoded shRNA (Supplementary Fig. 3B). There was no evidence of altered actin structure or cytoskeleton in these cells. CD16-Nephrin chimeras along with GFP-actin were expressed in the Ship2^kd^ Hupo and control cell lines. Upon Nephrin activation, we observed large aggregates of actin in Ship2^kd^ Hupo cells when compared to controls (Figure 7). In fact, Ship2 knock-down resulted in 65% of the cells exhibiting aggregates of actin greater than 3 µm in width compared to 20% in control (p<0.005) (Fig. 6C). Similarly, large actin aggregates were observed on Nephrin activation in transfected M2 cells. These aggregates were not observed when CD16-HA was clustered when Ship2 was knocked down or in the absence of Filamin (M2 cells). They were also not observed in the absence of aggregating antibody or in control experiment using CD16-HA (Figure S4), suggesting that the actin aggregates were generated on Nephrin activation and not as a result of lack of Ship2 or Filamin. To assure that these findings were specific to Ship2 or Filamin, we were able to rescue the phenotype by expressing rat Ship2 (Figure S3C), with sequence dissimilarity to human Ship2, in Ship2^kd^ Hupo cells, and by expressing Filamin in M2 cells. To assess whether abnormal actin aggregates are formed in the absence of Nephrin, we attempted to generate Nephrin knock-down podocyte cell lines. Even though the expression levels of Nephrin are extremely low in immortalized human podocytes, multiple attempts to generate Nephrin knock-down cell lines failed due to poor survival of cells following selection.

**Figure 8:**
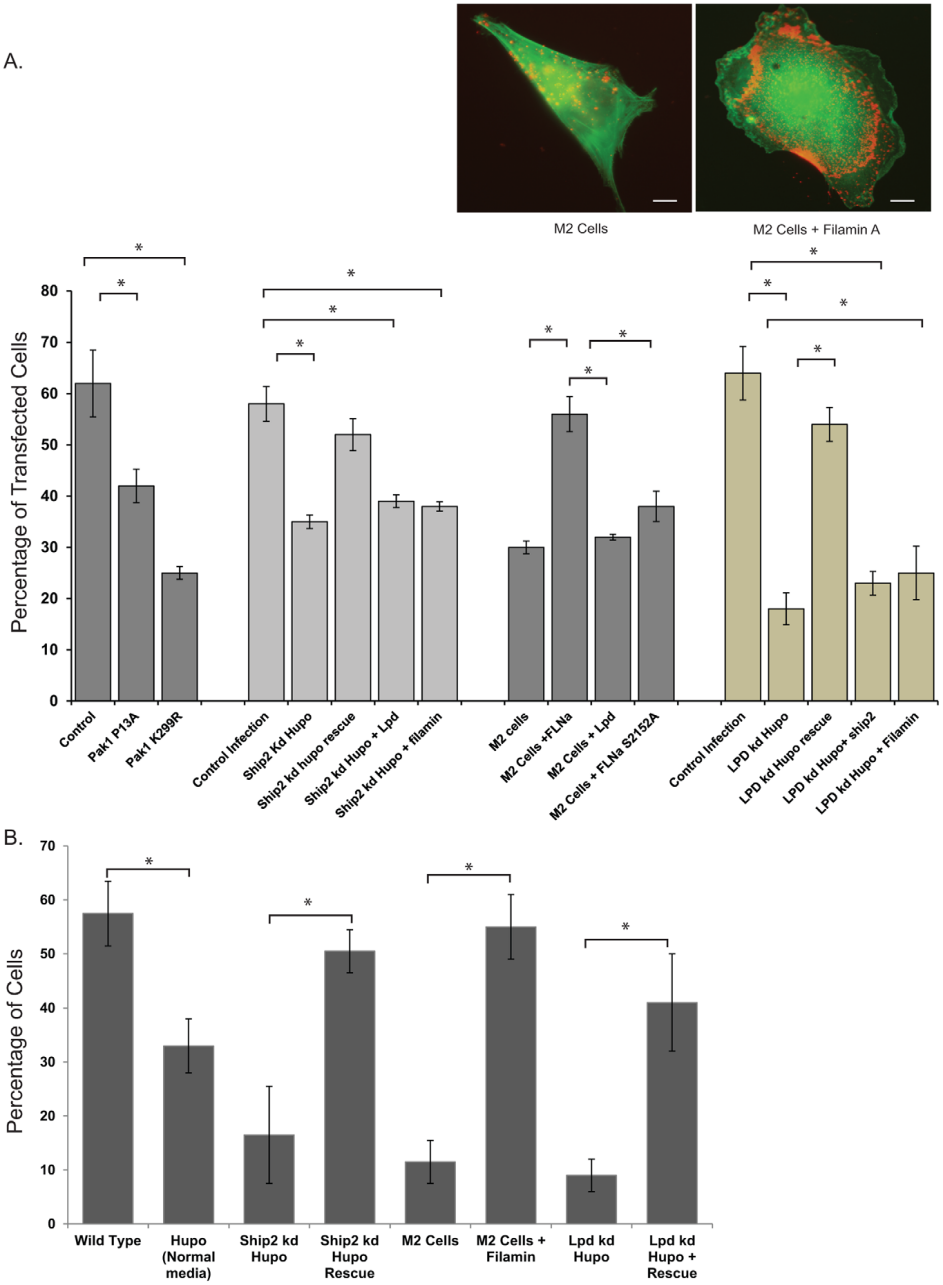
*A.* Nephrin dependent assembly of a protein complex that includes Ship2, Filamin and Lamellipodin are required for lamellipodia formation. **A.** *Lamellipodia formation following Nephrin activation in human podocytes (WT), Ship2^kd^ Hupo, LPD^kd^ Hupo and M2 cells*. Human podocytes expressing CD16-Nephrin chimeras were transfected with the indicated plasmids were clustered as described and examined by immunofluorescence microscopy. Two panels are representative cells from multiple experiments (Red: CD16 clusters; Green: GFP-Actin). At least 100 cells were examined in each condition. CD16-HA chimera was transfected in the control experiment. Results are shown as +/−SEM and are representative of four different experiments. * p<0.01. ***B.*** *Migration of cells using blind well chambers*. 100 µl of cell suspension containing human podocytes (WT), Ship2^kd^ Hupo, LPD^kd^ Hupo and M2 cells at 2000 cells/ml density was placed in the top chamber. Cells were counted on the underside of the filter following DAPI staining and the data represents percentage of cells compared to total cells pipetted into the well. Results are shown as +/−SEM and are representative of four different experiments. * p<0.01. Scale bar: 10 µm.

Based on formation of abnormal actin aggregates we further hypothesized that lamellipodia formation should also be affected in this model. We investigated the formation of lamellipodia when Ship2 or Filamin is knocked down or when Nephrin mediated Ship2 activation is perturbed by disrupting Pak1 activity. Clustered CD16-Nephrin expressing knock-down cell lines were stained with phalloidin to visualize actin. About 100 cells that were transfected based on CD16-neprhrin clusters at the membrane were counted for each experimental condition. All experiments were repeated at least 4 times for the final analysis. Clustering of CD16-Nephrin in human podocytes (control) cells resulted in approximately 62% of the cells showing lamellipodia like structures (see inset in Figure 8). In Ship2^kd^ Hupo cells, we observed a significant decrease (p<0.001) in the lamellipodia formation (Figure 8A) following CD16-Nephrin clustering. This phenotype was rescued by expressing rat Ship2 with sequence dissimilarity to human Ship2 (p-value NS). As expected, there was a decrease in lamellipodia formation in the M2 cell line that was rescued when wild type Filamin was expressed. There was only a partial rescue of lamellipodia formation with expression of the Filamin (S2152A) mutant that cannot be phosphorylated. A similar decrease in lamellipodia formation was also observed when the kinase dead Pak1 mutant (K299R) and Pak1 (P13A), a mutant that is unable to bind to Nck SH3 domain, were expressed.

To further extend our findings we generated a human podocyte cell line with LPD knock down (LPD^kd^ Hupo) using lentivirus mediated expression of shRNA (Figure S3D). We observed best knockdown using the shRNA #4 which is against a region in the 3′UTR of the LPD (RAPH1) gene. This knock down was rescued by expressing a Flag-LPD plasmid (Figure S3E). Lamellipodia formation, in response to activation of CD16-Nephrin, was less in the LPD^kd^ Hupo cells than in Ship2^kd^ Hupo cells (Figure 8A) and M2 cell lines (approx. 17%). To assess the furthest downstream protein responsible for the generation of lamellipodia as a result of Nephrin activation, we overexpressed LPD in Ship2^kd^ Hupo cells and M2 cells. There was only partial rescue of the diminished lamellipodia formation in Ship2^kd^ Hupo and M2 cell line with over-expression of LPD, these differences were statistically insignificant. Similarly, Filamin overexpression was also unable to completely rescue the phenotype in Ship2^kd^ Hupo cells. Interestingly, over-expression of Ship2 and Filamin in LPD^kd^ Hupo cells resulted in less lamellipodia formation when compared to the better partial rescue by expression of LPD in Ship2^kd^ Hupo and M2 cells. This observation would suggest recruitment of LPD is sufficient for Nephrin mediated lamellipodia formation under our experimental conditions. This would concur with the established role of LPD in focal adhesion dynamics and its role as a scaffold protein for assembling the protein complex responsible for lamellipodia formation [43].

To examine the consequence of lamellipodia formation on cell migration, we employed Boyden chambers to study migration of podocytes in response to 50A9 mab directed against human Nephrin extracellular domain. In concordance with our findings of lamellipodia formation, the number of cells that migrated across the filter was lower in M2 cells, Ship2 or LPD^kd^ Hupo cells when compared to control (Figure 8B).

Collectively, these results demonstrate the ability of Nephrin to assemble a protein complex that is responsible for lamellipodia formation. These findings also suggest the emerging role of Nephrin in regulation of the focal adhesion complex. Presumably these signaling events occur simultaneously and augment lamellipodia formation. In fact multiple pathways that might be relevant in unique contextual signaling events are able to generate lamellipodia formation on Nephrin activation.

## Discussion

The development of the podocyte foot process is likely to involve a yet unidentified polarized guidance cue that initiates the formation of a membrane protrusion by the nascent podocyte. It is assumed that the Nephrin-Neph1 complex is the transmembrane receptor that responds to the guidance cue. Initial investigations indicate that Nephrin assembles a protein complex that initiates actin polymerization presumably resulting in a membrane protrusion at the leading edge [7], [8], [15], [16]. To generate a complex membrane structure like lamellipodia, initial actin nucleation needs to be followed by elongation, branching and crosslinking of the actin filaments. In response to an undetermined guidance cue, the Nephrin-Neph1 complex is able to initiate polymerization of the actin filament in an Nck and Grb2 dependent fashion [7], [8], [16]. We have also demonstrated that Nephrin is able to activate and recruit cofilin, an actin depolymerizing protein that helps to replenish actin monomers and maintain the filament [15]. To further generate an actin network, Nephrin needs to assemble a whole gamut of actin related proteins that play a role in lamellipodia formation at the leading edge. Here we report that in cell culture, Nephrin assembles a protein complex that includes Ship2, Filamin and LPD in a tyrosine phosphorylation dependent manner resulting in lamellipodia formation. We also observe that in the absence of these proteins, following Nephrin activation there is an alteration of the morphology of the actin filament. Additionally, Nephrin's ability to form lamellipodia is diminished when Ship2, Filamin and LPD are knocked down in cultured cells. Taken together, these results suggest Nephrin is able to not only initiate actin polymerization but also assemble a protein complex that regulates the architecture of the actin network.

Furthermore, for a cell to be able to migrate or extend membrane processes in a polarized fashion it is essential for it to constantly generate and dismantle focal adhesions. Focal adhesions act as treads on which the cell moves and keeps in contact with the matrix. During cell migration, there is constant turnover of the focal adhesion complex requiring assembly and disassembly of a protein complex that include amongst other proteins integrins, focal adhesion kinase (FAK), Src kinases, p130cas, Ena/VASP and small GTPases (reviewed in [51], [52]). These sequences of events are required to be repeated multiple times by the cell to be able to respond to a guidance cue either for migrating as an individual cell or extending axons or dendrites, as in neurons, or processes, as in podocytes.

**Figure 9:**
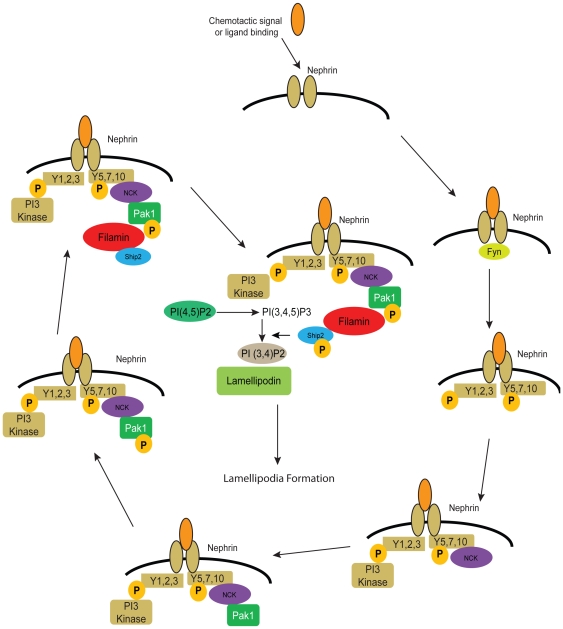
Schematic of proposed signaling. Nephrin activation results in *Src* kinase *Fyn* dependent phosphorylation on tyrosine residues as shown. Phosphorylation on Nephrin Y5,7,10 results in Nck dependent Pak1 recruitment. This further result in recruitment of the Filamin-Ship2 complex at the leading edge and is dependent on phosphorylation of Filamin S2152. Ship2 is enriched at the leading edge and dephosphorylates PI (3,4,5)P3 to PI (3,4)P2 and recruitment of Lamellipodin. It has been demonstrated previously by us and others that Nephrin recruits p85 subunit of PI3 kinase [10], [35].

During development, injury and subsequent repair, podocytes undergo robust cytoskeletal remodeling requiring highly regulated actin dynamics and focal adhesion turnover. In order for Nephrin to be able to regulate membrane protrusions in response to a polarized cue, it would need to regulate actin dynamics as well as focal adhesion turnover. Our identification of Nephrin's ability to recruit Ship2, Filamin and LPD, proteins that are not only important for actin dynamics and lamellipodia formation, but also focal adhesion regulation, demonstrates Nephrin's ability to regulate these processes (see Figure 9 for proposed signaling cascade). Ship2 is responsible for generation of PI(3,4)P2 at the membrane which has been implicated as one of the initial asymmetric signals at the leading edge [46]. Ship2 has also been shown to associate with the adapter protein p130cas and regulate cellular spreading and adhesion [53]. Filamin is an actin cross-linking protein and has been suggested to play a role in focal adhesion disassembly [21]. It acts as a negative regulator of integrin activation by blocking talin binding to integrin [54], [55]. This role is supported by studies where suppression of Filamin expression leads to increased invasiveness of human breast cancer cells [19], [20], [56] presumably by facilitating focal adhesion turnover. Filamin silencing also results in activation of calpain which leads to degradation of focal adhesion proteins [57].

Similarly, LPD interacts with the Ena/VASP proteins that are also an important component of the focal adhesion complex and for lamellipodia formation, though their role at the focal adhesion is not entirely clear. Lamellipodin also links PI-3 kinase signaling at the leading edge to effectors of the actin cytoskeleton. PI-3 kinase is recruited to the leading edge when cells are exposed to external chemotactic gradients [46], [58], [59], [60], [61]. In addition to the PH domains of TAPP1 and TAPP2 [42], LPD binds specifically to PI (3,4)P2 [43]. By facilitating generation of PI(3,4)P2, Nephrin is able to recruit Lamellipodin to the leading edge which then acts as a scaffold for proteins like Ena/VASP, further facilitating generation of lamellipodia. This is supported by our observation of decreased lamellipodia formation following Nephrin activation in cells where Ship2, Filamin or LPD are knocked down. Since lamellipodia formation in Ship2^kd^ Hupo and M2 cells can be restored by overexpressing Lamellipodin, suggesting that Lamellipodin recruitment to the leading edge is sufficient to generate a lamellipodia.

Our observations further extend the understanding of the complex assembled by Nephrin in order for it to regulate actin dynamics in podocytes that occur both during development and following injury. These findings demonstrate Nephrin's ability to not only initiate the generation of the actin filament but also regulate the architecture of the actin network thus generated. One would presume that these events would occur simultaneously in a coordinated manner but at present that cannot be completely resolved based on our model system. Whether in nature the signaling events described occur exclusively upon Nephrin activation is also difficult to study because of the existing limitations in available model systems and technology.

## Supporting Information

Figure S1 **Nephrin recruits Nck.** Human podocytes expressing GFP-Nck were transfected with CD16-HA (con) or CD16-Nephrin. Following clustering as described cells were examined by confocal microscopy. Scale bar: 10 µm.
(TIF)

Figure S2 **Pak1 is necessary for Nephrin-Filamin recruitment.** *Pak1 knock-down human podocytes.* Using shRNA mediated knock-down we generated 5 different cell lines. ShRNA#3 had optimal Pak1 knock down and were used for the experiment. Control vector had a non-targeting sequence. Cell lysates were blotted with antibodies against pak1 and actin. **B.** *Pak1 is necessary for recruitment of Filamin to CD16-Neprhin clusters*. Human podocytes with stable integration of indicated Pak1GFP-shRNA were transfected with CD16-Nephrin and DsRed-Filamin. Clustering of CD16 chimera was performed as described and cells were examined using immunofluorescence microscopy. Scale bar: 10 µm.
(TIF)

Figure S3 **Knock-down Cell Lines.** **A.** *Expression of Filamin in M2 cell line*. Lysates from M2 cells, Human podocytes (Hupo) and M2 cells transfected with Filamin were blotted for expression of Filamin. **B.** *Ship2^kd^ Hupo cell line*. Expression of Ship2 in stable cell lines following lentivirus infection containing five different shRNA. Cells incorporating shRNA #2 was used for additional studies. Lysates from breast cancer cell line MDA-MB231 that express high levels of endogenous Ship2 was used as control. **C.** *Ship2^kd^ Hupo Rescue*. Rat Ship2 with sequence dissimilarity to human Ship2 was expressed to rescue Ship2 in knock down cell line. Lysates from the cell lines were examined for Ship2 expression. **D.** *Lamellipodin knock down cell line (LPD^kd^ Hupo)*. Human podocyte cell lines with stable integration of five different shRNA were produced. Cell lysates were examined for LPD expression. Cells incorporating shRNA #4 was selected for additional studies. **E.** *LPD^kd^ Hupo rescue*. Flag-LPD expressing human LPD was used to rescue LPD in knock-down cell line. LPD shRNA #4 is directed against a 3′UTR region in human LPD gene.
(TIF)

Figure S4 **Ship2^kd^ Hupo and M2 cells have normal actin cytoskeleton.** Ship2^kd^ Hupo and M2 cells expressing RFP- Actin were transfected with CD16-Nephrin and CD16-HA. Actin cytoskeleton was normal in morphology in the absence of CD16-Nephrin clustering or when cells transfecting with CD16-HA were clustered.
(TIF)
