# Supplementary figures and images for: Nephrin Regulates Lamellipodia Formation by Assembling a Protein Complex That Includes Ship2, Filamin and Lamellipodin
Source: PLoS One. 2011 Dec 14;6(12):e28710. doi: 10.1371/journal.pone.0028710 (PMC3237483; doi:10.1371/journal.pone.0028710)

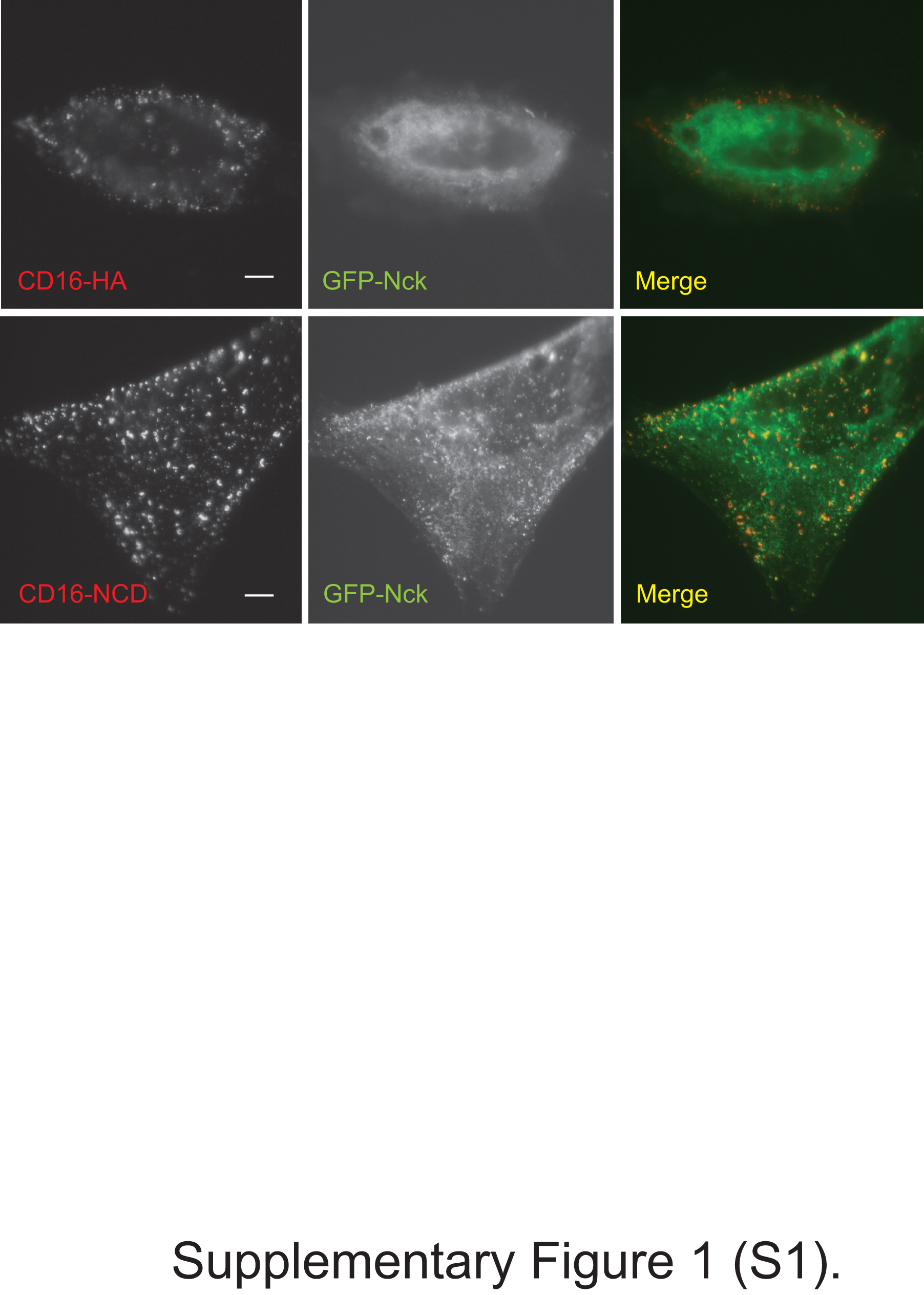

Supplement: Figure S1 — Nephrin recruits Nck. Human podocytes expressing GFP-Nck were transfected with CD16-HA (con) or CD16-Nephrin. Following clustering as described cells were examined by confocal microscopy. Scale bar: 10 µm. (TIF) [file pone.0028710.s001.tif]

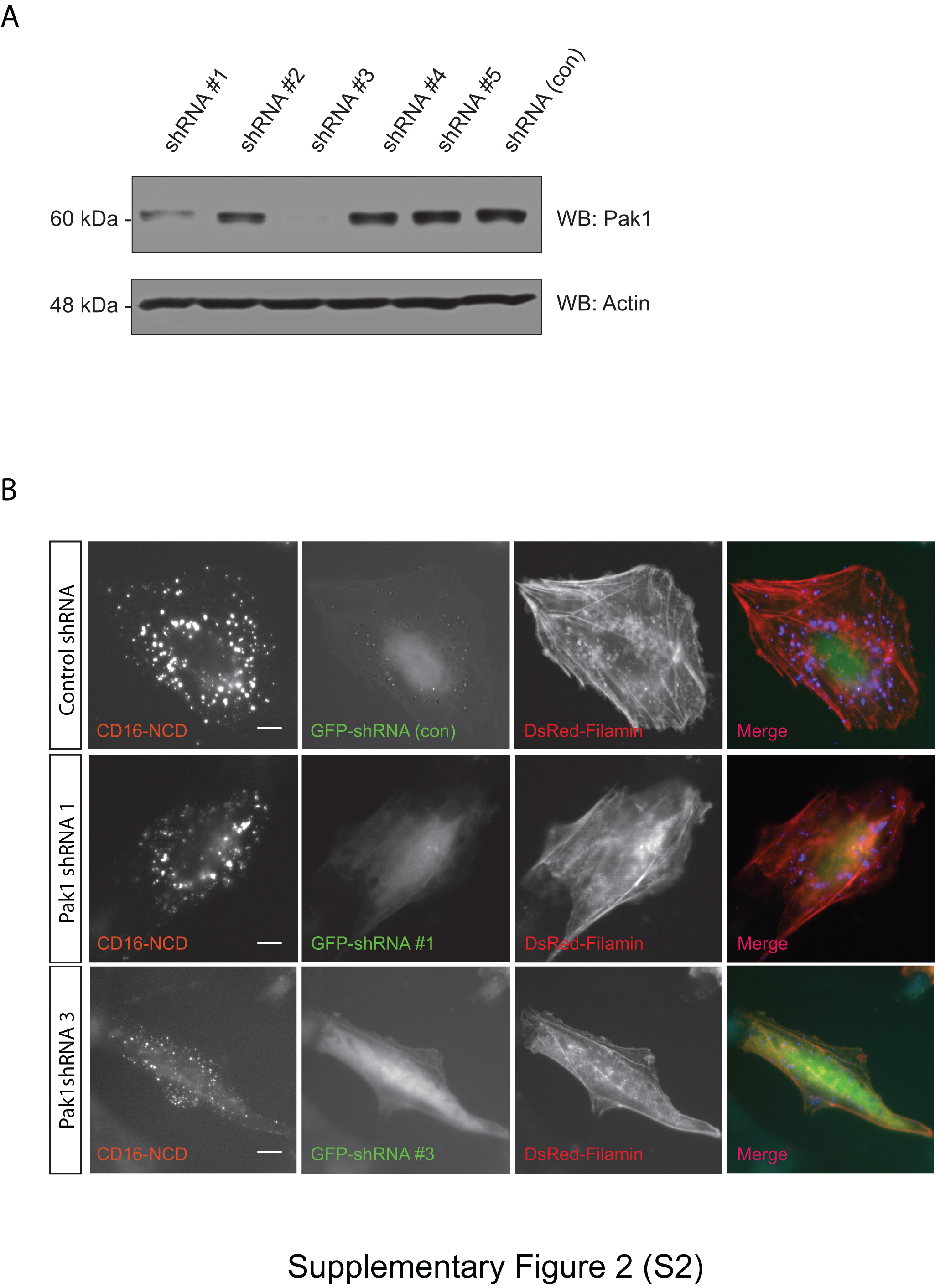

Supplement: Figure S2 — Pak1 is necessary for Nephrin-Filamin recruitment. Pak1 knock-down human podocytes. Using shRNA mediated knock-down we generated 5 different cell lines. ShRNA#3 had optimal Pak1 knock down and were used for the experiment. Control vector had a non-targeting sequence. Cell lysates were blotted with antibodies against pak1 and actin. B. Pak1 is necessary for recruitment of Filamin to CD16-Neprhin clusters. Human podocytes with stable integration of indicated Pak1GFP-shRNA were transfected with CD16-Nephrin and DsRed-Filamin. Clustering of CD16 chimera was performed as described and cells were examined using immunofluorescence microscopy. Scale bar: 10 µm. (TIF) [file pone.0028710.s002.tif]

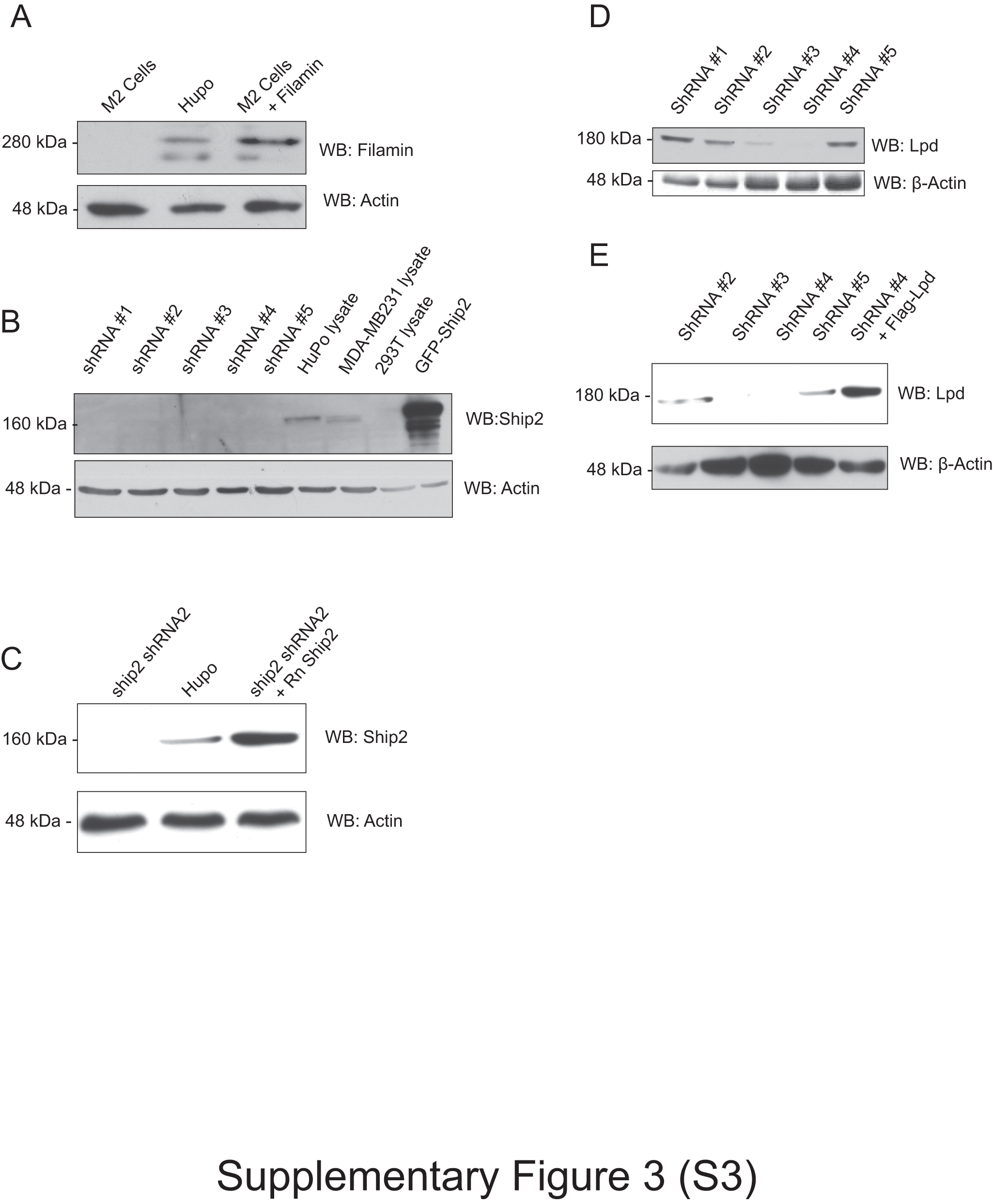

Supplement: Figure S3 — Knock-down Cell Lines. A. Expression of Filamin in M2 cell line. Lysates from M2 cells, Human podocytes (Hupo) and M2 cells transfected with Filamin were blotted for expression of Filamin. B. Ship2kd Hupo cell line. Expression of Ship2 in stable cell lines following lentivirus infection containing five different shRNA. Cells incorporating shRNA #2 was used for additional studies. Lysates from breast cancer cell line MDA-MB231 that express high levels of endogenous Ship2 was used as control. C. Ship2kd Hupo Rescue. Rat Ship2 with sequence dissimilarity to human Ship2 was expressed to rescue Ship2 in knock down cell line. Lysates from the cell lines were examined for Ship2 expression. D. Lamellipodin knock down cell line (LPDkd Hupo). Human podocyte cell lines with stable integration of five different shRNA were produced. Cell lysates were examined for LPD expression. Cells incorporating shRNA #4 was selected for additional studies. E. LPDkd Hupo rescue. Flag-LPD expressing human LPD was used to rescue LPD in knock-down cell line. LPD shRNA #4 is directed against a 3′UTR region in human LPD gene. (TIF) [file pone.0028710.s003.tif]

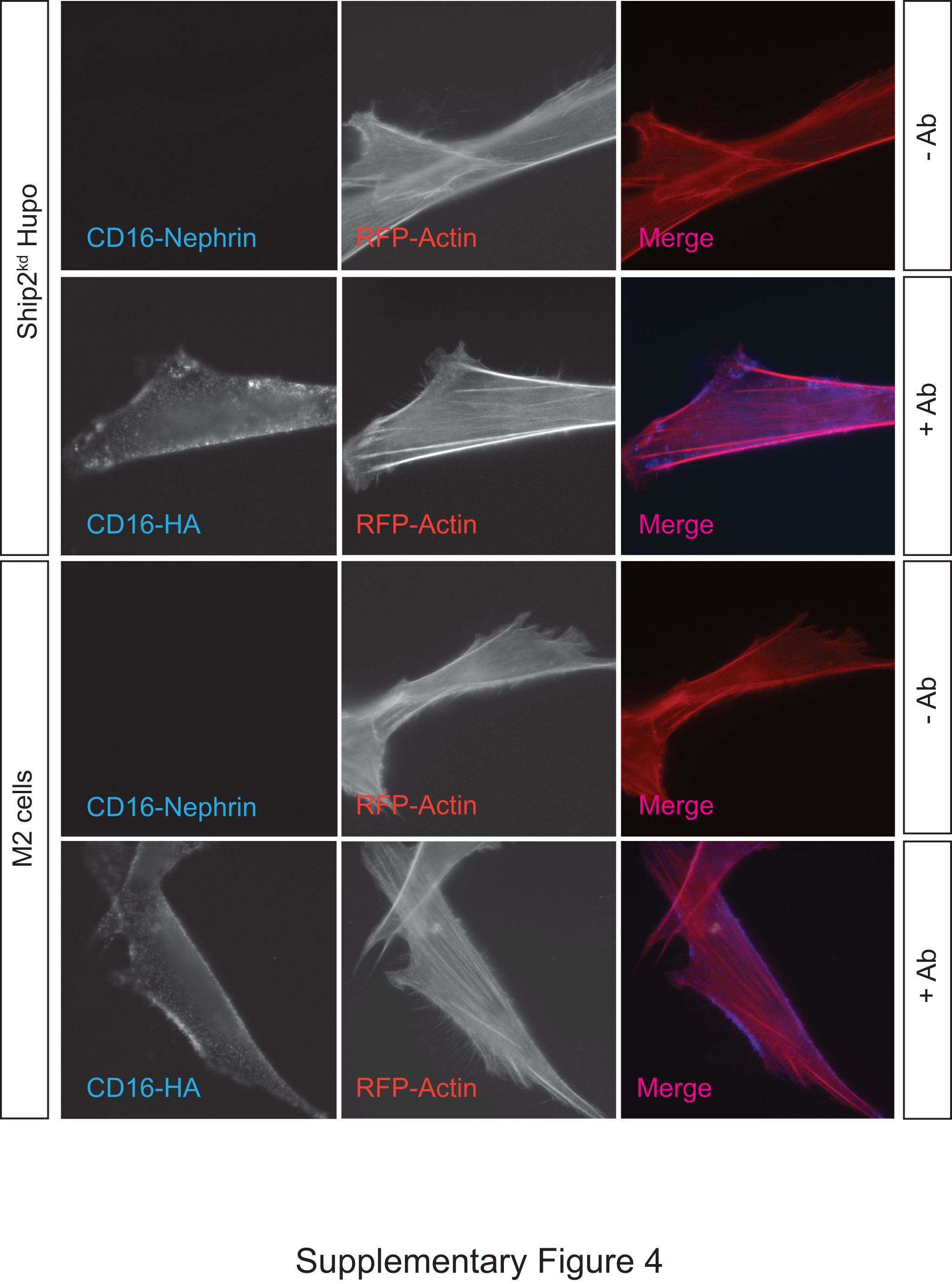

Supplement: Figure S4 — Ship2kd Hupo and M2 cells have normal actin cytoskeleton. Ship2kd Hupo and M2 cells expressing RFP- Actin were transfected with CD16-Nephrin and CD16-HA. Actin cytoskeleton was normal in morphology in the absence of CD16-Nephrin clustering or when cells transfecting with CD16-HA were clustered. (TIF) [file pone.0028710.s004.tif]
